# Comparison of Utero-Placental Perfusion Development From First to Second Trimester Between Low-Risk and High-Risk Pre-eclampsia Groups With Aspirin Prophylaxis

**DOI:** 10.7759/cureus.62309

**Published:** 2024-06-13

**Authors:** Jochen Ritgen, Julia Roxin, Marit Kolsch, Arne Bergsch, Jan Degenhardt

**Affiliations:** 1 Center for Prenatal Medicine and Genetics, Praenatal Plus, Cologne, DEU; 2 Medical Department, Justus-Liebig-University, Giessen, DEU

**Keywords:** uterine artery, aspirin, doppler ultrasound, prophylaxis, acetylsalicylic acid, prenatal ultrasound, preeclampsia, uteroplacental perfusion

## Abstract

Introduction

Pre-eclampsia (PE) is a common diagnosis in pregnancy and affects pregnancies worldwide. Early-onset PE often leads to severe maternal and fetal complications. Prophylactic use of aspirin (150 mg/day) before the 16th week of pregnancy can reduce the risk of PE. This study aimed to investigate the effects of maternal factors on the development of uteroplacental perfusion and fetal biometry from the first to the second trimester in a risk group receiving aspirin prophylaxis compared to a control group without Aspirin.

Methods

This case-control study included 448 women at high risk for PE (risk group, RG) receiving aspirin prophylaxis and 468 women at low PE risk without aspirin intake (control group, CG). Parameters recorded and considered in the first (T1) and second (T2) trimesters included uterine artery pulsatility multiple of the median (UtAPI MoM), notching at T1 and T2 and fetal biometry parameters at T2. Maternal factors were also captured, and their respective effects were examined.

Results

UtAPI MoM at T1 and T2 showed a significant positive correlation (r = 0.39, p < 0.001), with UtAPI MoM at T2 significantly higher for notching "yes" at T1. Pre-existing arterial hypertension and UtAPI development demonstrated a significant association (p = 0.006). Women without this risk factor showed a significantly (p < 0.001) greater decline in UtAPI development. The likelihood of notching "yes" at T2 (p < 0.001; OR: 5.80) was increased with higher UtAPI MoM at T1. The mean values (T1 and T2) of UtAPI MoM were significantly higher in the risk group than in the control group. Patients in the risk group exhibited notching at T2 (p < 0.001; OR: 5.64) more often compared to the control group. The 95% CI of the estimated fetal weight for notching "yes" at T1 was below the 50th percentile. Gestational age and head circumference/abdomen circumference (HC/AC) ratio showed a significant negative correlation (p < 0.001; b = -0.01). The control group showed significantly higher estimated fetal weights than the risk group. The HC/AC ratio in the risk group was above the HC/AC ratio in the control group but without proving significance.

Conclusions

Persistent notching and elevated UtAPI MoM levels in the second trimester may be risk factors for early-onset PE. Women with pre-existing arterial hypertension, notching and elevated UtAPI MoM values ​​in the first and second trimesters require special monitoring during the course of pregnancy.

## Introduction

Preeclampsia is a progressive disorder affecting multiple organ systems and affects 2-8% of pregnancies [1]. It is marked by the sudden onset of high blood pressure and proteinuria, or the sudden onset of high blood pressure combined with significant dysfunction of internal organs, with or without proteinuria. Typically, it presents after 20 weeks of gestation or postpartum. The pathogenesis involves both abnormal placentation and maternal systemic vascular dysfunction. While maternal risks are mainly related to the severity of maternal symptoms, fetal risks are closely related to prematurity and placental insufficiency. The primary goal of risk assessment in the first trimester is to identify patients at high risk (> 1:100) for developing preeclampsia requiring delivery before 34 weeks who would benefit from prophylaxis using acetylsalicylic acid. The individual risk is assessed in the first trimester based on maternal characteristics, medical history, along with biophysical factors (adjusted pulsatility index of the uterine arteries, mean arterial blood pressure) and biochemical measurements (such as pregnancy-associated plasma protein A (PAPP-A) and placental growth factor (PlGF)). This risk assessment takes into account the major risk factors for preeclampsia that lead to the recommendation of aspirin in pregnancy, considering frequently used guidelines [2,3]. In the ASPRE (Aspirin for Evidence-Based Preeclampsia Prevention) study, women with a risk > 1:100 for early PE were randomized into an aspirin arm and a placebo arm. The study showed that taking aspirin (150 mg/d) reduced the PE rate by up to 60% [4]. This effect is believed to be caused by the prevention of platelet aggregation and by altering platelet prostaglandin synthesis [5].

Objectives

In the present study, we investigate the development of uteroplacental perfusion from the first to the second trimester within a group at high risk for preterm preeclampsia (PE) under aspirin prophylaxis. Another objective of this study is to examine the associations between uteroplacental perfusion in the first trimester and fetal biometry in the second trimester within the risk group. Additionally, we aim to assess the relationships and effects of maternal factors such as pre-existing arterial hypertension, nicotine consumption during pregnancy, body mass index (BMI), and gestational age on the development of uteroplacental perfusion and fetal biometry to explore their predictive value. Furthermore, this study aims to compare the developments and associations of uteroplacental perfusion from the first trimester to the second trimester between pregnant women at high risk for PE receiving aspirin prophylaxis and pregnant women at low risk for PE. Differences between these two groups were analyzed.

This article was previously presented as a meeting abstract at the 43rd Meeting of the German Society for Ultrasound in Medicine (DEGUM), the Austrian Society for Ultrasound in Medicine (OEGUM), and the Swiss Society for Ultrasound in Medicine (SGUM) on October 19, 2019.

## Materials and methods

This case-control study included women who participated in a first-trimester screening (T1) between 11+0 and 13+6 weeks of gestation. Additionally, they underwent a second ultrasound examination during the second trimester (T2) between 15+0 and 26+6 weeks of gestation as part of a detailed fetal anomaly scan. Ultrasound measurements were performed between April 2012 and February 2019 at the Center for Prenatal Medicine and Genetics, Praenatal Plus, in Cologne.

The study’s inclusion criteria were pregnant women who had reached the age of 18 or older, had singleton pregnancies and had live fetuses between 11+0 and 13+6 weeks of gestation. Women with multiple pregnancies, pregnancy terminations, intrauterine fetal demise before 24 weeks of gestation, aspirin intolerance, and pregnancies with significant fetal anomalies or chromosomal aberrations were excluded from the study. During first-trimester screening (FTS), an individualized risk for developing preeclampsia (PE) before 34 weeks of gestation was determined using the screening algorithm developed by the Fetal Medicine Foundation (FMF) London [6]. Based on this risk assessment, two patient groups were formed: Risk Group (RG): This group included 448 pregnant women at an increased risk for developing PE before 34 weeks of gestation (risk > 1:100 according to the FMF screening algorithm). Control Group (CG): This group comprised 468 pregnant women with an unremarkable PE risk screening, selected as a random sample for comparison.

Detailed information on the characteristics of patient groups is provided in Tables 1, 2. Pregnant women in the risk group received a daily dose of 100 mg aspirin for PE prophylaxis from the time of screening. During the study period, the regimen changed to 150 mg from September 2017 onwards. We considered this change appropriate in order to adapt to the dosage used in the ASPRE study [4].

**Table 1:** Maternal physical characteristics Weight, BMI and gestational age are described for T1 and T2.

Maternal Characteristics	Risk Group (n = 448)	Control Group (n =468)
	Mean ±SD	Mean ±SD
Maternal age, years	34.6 ±4.3	34.6 ±4.3
Maternal height, cm	166.6 ±6.2	168.8 ±6.6
Maternal weight at T1, kg	73.3 ±17.5	68.6 ±12.2
Maternal weight at T2, kg	76.0 ±17.2	71.6 ±12.2
Maternal BMI at T1, kg/m²	26.4 ±5.8	24.1 ±4.1
Maternal BMI at T2, kg /m²	27.3 ±5.7	25.1 ±4.1
Gestational age at T1, weeks	12.7 ±0.5	12.6 ±0.5
Gestational age at T2, weeks	21.1 ±1.3	21.3 ±1.2

**Table 2:** Maternal characteristics: Ethnicity, conception, nicotine consumption, pre-existing medical conditions, parity and positive family history of PE

Maternal Characteristics	Risk Group (n = 448)		Control Group (n = 468)	
	n	%	n	%
Ethnicity				
East Asian	5	1.1	5	1.1
Black	0	0	2	0.4
South Asian	3	0.7	2	0.4
White	433	96.7	431	92.1
Black-White	1	0.2	1	0.2
South Asian - East Asian	1	0.2	0	0
Missing data	5	1.1	27	5.8
Conception				
Artificial insemination by donor/husband (AID/AIH)	4	0.9	2	0.4
Intracytoplasmic sperm injection (ICSI)	40	8.9	20	4.3
in vitro fertilization (IVF)	17	3.8	7	1.5
Ovulation induction	0	0	1	0.2
Natural conception	387	86.4	436	93.2
Missing data	0	0	2	0.4
Nicotine consumption				
smoking	16	3.6	15	3.2
Pre-existing medical conditions				
Arterial hypertension	72	16.1	3	0.6
Diabetes mellitus Type 1	8	1.8	4	0.9
Diabetes mellitus Type 2	6	1.3	2	0.4
Antiphospholipid Syndrome	7	1.6	1	0.2
Systemic Lupus erythematodes	1	0.2	0	0
Parity				
Nullipara	300	67	225	48.1
Para with history of PE	67	15	10	2.1
Para without a history of PE	78	17.4	233	49.8
Family history				
Positive famiiy history of PE	21	4.7	2	0.4

The maternal mean arterial blood pressure (MAP) was recorded at T1 according to the FMF guidelines. The MAP is defined as the average arterial blood pressure during one cardiac cycle. The average of the four measurements is reported in mmHg and adjusted for maternal factors in multiples of the median (MoM) values. Additionally, at T1, biochemical laboratory values from maternal serum were determined as part of the FTS. Serum markers included pregnancy-associated plasma protein-A (PAPP-A) and placental growth factor (PlGF). Concentrations were determined rapidly using a fully automated analyzer (Brahms Kryptor, Thermo Fisher Scientific, Henningsdorf, Germany) and converted to MoM values.

Ultrasound examinations of the uterine arteries (Aa. uterinae) were performed at both T1 and T2. These transabdominal ultrasound scans were conducted by qualified examiners (German Society for Ultrasound in Medicine grade II or III certification) using devices such as Aplio 500 and Aplio i900 (Canon Medical, Neuss, Germany), Voluson E10 (GE Healthcare, Solingen, Germany) and Epiq 7 (Philips Medical Systems, Netherlands). Doppler flow curves were examined for the presence of notching and the pulsatility index (PI). The PI is calculated by the difference between the maximum systolic velocity (S) and the minimum diastolic flow velocity (D), divided by the mean flow velocity (Vm): PI = (S-D)/Vm. The mean uterine artery pulsatility (UtAPI) was derived from the PI of both Aa. uterinae (left and right uterine artery).

Fetal biometry

As part of the FTS, the crown-rump length (CRL) was measured transabdominally. The SSL was measured with the fetus in a neutral position. At T2, the biparietal diameter (BPD) and the fronto-occipital diameter (FOD), the abdominal circumference (AC), and the femur length (FL) were measured. The head circumference/abdomen circumference (HC/AC) quotient was calculated. The estimated fetal weight (EFW) was calculated using Hadlock's formula.

Statistical analysis

The data entry was initially done in Microsoft Excel and was quality-controlled. The statistical analysis and graphical representation were performed after statistical consultation using STATA software, version 16 (StataCorp LLC., College Station, TX). In descriptive statistics, categorical scaled variables were presented in absolute and relative frequencies. For metric variables, measures of central tendency (median, mean) and dispersion (maximum, minimum, standard deviation, interquartile range) were calculated.

An exploratory approach was chosen to depict and analyze the associations between individual variables related to uteroplacental perfusion at two time points and fetal biometry variables. To account for the effects of maternal factors on the dependent variables, maternal factors were included as covariates in the analyses. Throughout the entire study, an alpha error level of 0.05 was selected, and thus, all p-values < 0.05 were interpreted as significant.

In the first approach, the qualitative (notching) and quantitative uterine artery pulsatility multiple of the median (UtAPI MoM) uteroplacental perfusion at time point T1 within the risk group was compared with the uteroplacental perfusion (notching and UtAPI MoM) at time point T2. The association between notching at T1 and UtAPI MoM at T2 was assessed using linear regression analysis. The UtAPI MoM values at T2 were transformed (natural logarithm) before the analysis to achieve an approximate normal distribution. For the assessment of the categorical variable “notching” (yes, indicated, no) at both time points T1 and T2, descriptive statistics were used.

To analyze the development of UtAPI MoM values from T1 to T2, the difference method (DT2-T1) was employed, as the UtAPI MoM values at T1 had different baseline levels. The development of UtAPI MoM values was calculated as the individual difference (DT2-T1) and served as the dependent variable. The Pearson correlation coefficient was computed for this analysis. Due to heterogeneity of variance, robust standard errors were requested when analyzing the influences of maternal factors.

Multinomial logistic regression was used to assess the relationships between UtAPI MoM at T1 and the categorical scaled variable “notching” (yes, indicated, no) at T2. Odds ratios were calculated, and entry probabilities were graphically displayed for better and more accurate visualization.

Additionally, the relationships between qualitative and quantitative uteroplacental perfusion and fetal biometric variables EFW (percentile and grams) as well as HC/AC quotient were examined. Multiple linear regression analyses were used to assess the associations between UtAPI MoM at T1 and EFW in grams at T2, with robust standard errors requested due to variance heterogeneity. To emphasize and confirm the tested associations within the RG, further analyses were conducted. In these analyses, the RG and CG were compared, and the differences between the RG and CG were presented.

Linear regression analysis was used to examine the relationship between quantitative uteroplacental perfusion from T1 to T2, as well as the influences of maternal factors. The mean UtAPI MoM at T2 was transformed (natural logarithm) before analysis to achieve an approximate normal distribution. In the analyses between the two groups, the association between maternal factors and the development of UtAPI MoM values, as well as between notching T1 and UtAPI MoM development was examined. The development of UtAPI MoM values was assessed using the difference variable (DT2-T1). The associations were tested using linear regression analysis.

Multinomial logistic regression analysis was used to examine the associations between the groups (RG vs. CG) and the dependent interval scaled variable notching T2, as well as between UtAPI MoM values at T1 and notching at T2. For comparisons and differences between the RG and CG regarding uteroplacental perfusion and fetal biometry, multiple linear regression analyses were applied. Interactions between the various independent variables and dependent variables were considered and modeled in relation to the tested associations. When interactions between the groups were found to be nonsignificant in the tested associations, the values were centered and main effects were reported for both groups. The Justus Liebig University Ethics Committee in Giessen approved the study protocol (Reference: AZ89/21).

## Results

The composition of the study group is shown in Tables 1, 2. Nine hundred and sixteen patients (448 risk group and 468 control group) were included in the statistical analysis.

Uteroplacental perfusion within the study group

The uteroplacental perfusion changes from the first trimester to the second trimester. The relationship between the notching at T1 and the UtAPI MoM values at T2 was investigated using analysis of covariance (ANCOVA). Covariates included the BMI of women at T2, nicotine consumption (yes/no), and pre-existing arterial hypertension (yes/no). While the estimated mean values of UtAPI MoM at T2 for the notching groups at T1 “no” and “indicated” hardly differed, the mean values of UtAPI MoM at T2 for the notching group at T1 “yes” were significantly higher (p < 0.001). The relationship between UtAPI MoM at T1 and UtAPI MoM at T2 showed a positive significant correlation (r = 0.39; p < 0.001). It was found that the effects of gestational age at T1, nicotine, and BMI were not significantly different. However, a significant association was observed between pre-existing arterial hypertension and the development of UtAPI MoM values (p = 0.006). For cases without pre-existing arterial hypertension, compared to those with pre-existing arterial hypertension, there was a significantly greater decrease in UtAPI MoM values. The mean decrease in values was significant for the group without pre-existing arterial hypertension (p < 0.001), while it could not be statistically confirmed for the group with pre-existing arterial hypertension (p = 0.053). The relationship between nicotine consumption and the development of UtAPI MoM values was not statistically significant (p = 0.594). Additionally, the "notching yes" group at T2 already had higher UtAPI MoM values at T1. Higher UtAPI MoM values at T1 were associated with an increased likelihood of "notching yes" (p < 0.001; OR: 5.80) and "notching indicated" (p = 0.003; OR: 4.24) at T2.

Comparison of uteroplacental perfusion between groups

An interaction between the two groups (CG and RG) and UtAPI MoM T1 was found to be nonsignificant (p = 0.621). Additionally, the covariates of nicotine consumption (p = 0.593) and preexisting arterial hypertension (p = 0.436) showed no statistically significant associations with UtAPI MoM T2.

**Figure 1:**
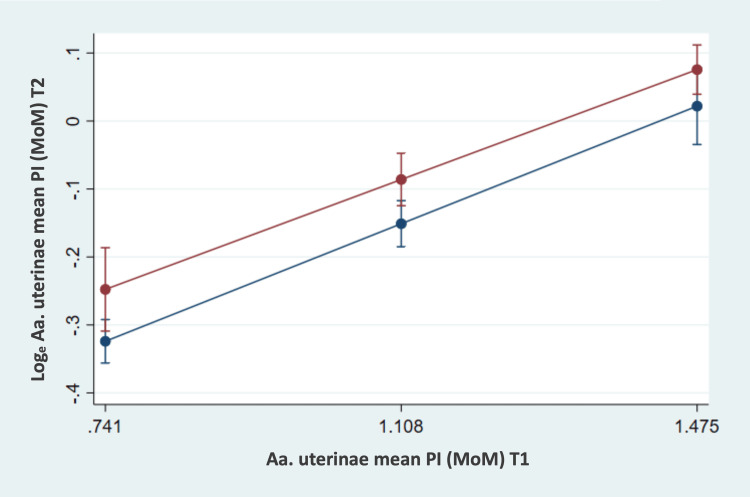
Uterine artery perfusion development between T1 and T2 The risk group (red) and control group (blue) are compared. Log_e_ uterine artery pulsatility multiple of the median (UtAPI MoM) at T2 (bars represent 95% confidence intervals) and UtAPI MoM at T1 (estimated mean ± Standard deviation). Aa. uterinae: Ultrasound examinations of the uterine arteries.

The relationship between UtAPI MoM values at T1 and UtAPI MoM values at T2 was highly significant (b = 0.47; p < 0.001). The higher the UtAPI MoM at T1, the higher the UtAPI MoM value remained at T2 (Figure 1). The mean UtAPI MoM values at T1 and T2 were slightly significantly higher in the RG compared to the CG (p = 0.044).

The significant association between preexisting arterial hypertension and UtAPI MoM development revealed a stronger decline in UtAPI MoM values between the time points in the subgroup without preexisting arterial hypertension. The notching groups at T1 differed significantly in the development of UtAPI MoM values when considering both groups. The decline in UtAPI MoM values was much stronger and highly significant (p < 0.001) for notching “yes” compared to notching “indicated“ or “no” at T1 (Table 3).

**Table 3:** Relationship between notching at time 1 (T1) and the pulsatility index of Aa. uterinae (UtAPI MoM) at time 2 (T2) UtAPI MoM: Uterine artery pulsatility multiple of the median, Aa. uterinae: Ultrasound examinations of the uterine arteries

Aa. uterinae Notching T1, n = 417, R^2 ^= 0.09	Log_e_ mean values UtAPI MoM T2	95 % confidence interval	UtAPI MoM T2	p-value	
				no	indicated
No (n = 96)	- 0.15	- 0.22, - 0.08	0.86		
Indicated (n = 25)	- 0.13	-0.26, - 0.00	0.88	1	
Yes (n = 296)	0.07	0.03, 0.11	1.07	< 0.001	0.010

Regarding the relationship between the covariate BMI at T1 and the development of UtAPI MoM values, a slightly significant interaction (p = 0.026) was observed between the RG and CG. The development of UtAPI MoM values differed significantly between the RG and CG based on BMI at T1. At a low BMI of 20 kg/m², the CG and RG differed only slightly and nonsignificantly (p = 0.159) in UtAPI MoM development. However, at moderate and higher BMIs (25.1 kg/m²; 30.2 kg/m²), the CG and RG differed significantly (p < 0.001; p < 0.001) from each other. The higher the BMI, the greater the difference in UtAPI MoM development between the two time points within the RG and CG. Consistently higher UtAPI MoM values were observed at T1 within the RG.

Relationship between uteroplacental perfusion and biometric parameters

Table 4 displays the relationships between uteroplacental perfusion (notching T1 and UtAPI MoM T1) and the head circumference/abdominal circumference ratio T2.

**Table 4:** Relationships between uteroplacental perfusion (notching T1 and UtAPI MoM at T1), gestational age, medical history and the HC/AC ratio at T2 UtAPI MoM: Uterine artery pulsatility multiple of the median; HC: Head circumference; AC: Abdomen circumference, GA: Gestation age

R^2 ^= 0.10, n = 427	Head-/Abdomen-Circumference (HC/AC) Quotient T2	
	p	mean HC/AC T2 (95 % CI)
Notching T1		
No (n = 100)	0.628	1.15 (1.14, 1.16)
Indicated (n = 27)		1.14 (1.12, 1.16)
Yes (n = 300)		1.15 (1.14, 1.16)
UtAPI MoM T1	0.542	
Body Mass Index T2	0.813	
GA T2	< 0.001	
Nicotine		
No (n = 411)	0.133	1.15 (1.15 – 1.16)
Yes (n = 16)		1.13 (1.11 – 1.16)
Preexisting arterial hypertension		
No (n = 381)	0.112	1.15 (1.15 – 1.16)
Yes (n = 46)		1.14 (1.12 – 1.15)

The estimated fetal weight differed between the RG and CG at T2. The CG had significantly larger EFW percentile values at T2 compared to the RG (p = 0.027). No significant difference was found in the relationship between UtAPI MoM T1 values and the HC/AC ratio at T2 between the RG and CG. Although the HC/AC ratio in the RG was higher overall than in the CG, it did not reach statistical significance (p = 0.121).

The association between UtAPI MoM T1 values and EFW percentile at T2 showed a slight significance (p = 0.028). However, the interaction between the RG and CG regarding the relationship between UtAPI MoM T1 values and RG EFW percentile at T2 was not statistically confirmed (p = 0.131). Similarly, there was no significant association (p = 0.063) or interaction (p = 0.120) between UtAPI MoM T1 values and the HC/AC ratio at T2 within the CG and RG.

Given the nonsignificant interactions between the groups, the values were centered, and the main effects were examined. Statistically significant negative correlations were observed between gestational age (GA) at T2 and the EFW percentile at T2, as well as the HC/AC ratio at T2, in both the CG and RG. Additionally, a statistically supported positive correlation was found between BMI at T2 and EFW percentile at T2.

## Discussion

Our study focuses on early utero-placental vascular development and its transition toward the second trimester. The pathophysiological changes in the placenta in cases at risk of early-onset PE occur very early in pregnancy. Prophylactic use of aspirin (150 mg/day) reduces the risk of PE when started before the 16th week of gestation [7]. The link between PE and increased platelet turnover and elevated levels of platelet-derived thromboxane provided the impetus for randomized trials assessing the efficacy of low-dose aspirin in high-risk patients. Unlike higher doses, low-dose aspirin reduces thromboxane production by platelets without significantly affecting prostacyclin synthesis by the vascular endothelium [5]. Meta-analyses of randomized trials offer substantial evidence supporting the prophylactic use of low-dose aspirin in pregnancy to modestly decrease the risk of pre-eclampsia and related complications. For instance, a 2019 meta-analysis encompassing 74 trials and over 40,000 participants across varying risk categories revealed several key outcomes when comparing low-dose aspirin (ranging from 50 to 162 mg per day) to placebo or no treatment (a reduction in cases of pre-eclampsia, a decrease in fetal or neonatal death, a reduction in preterm births before 37 weeks, a decrease in the incidence of small-for-gestational-age newborns, and a reduction in serious composite adverse maternal and neonatal outcomes [1].

Despite the risk assessment in the first trimester and identifying women who would benefit from aspirin treatment, further evaluations during the second and third trimesters remain important. Examinations during the second and third trimesters aim to reassess the patient-specific risk for the development of pre-eclampsia (PE). Studies have demonstrated an 85% detection rate for the development of PE before 37 weeks of gestation, with a false-positive rate of 10% [8,9]. 

In normal pregnancies, the resistance in the Aa. uterinae decreases physiologically over the course of pregnancy from weeks 11 to 34 of pregnancy [10]. The decrease in UtAPI from the first to the second trimester is steeper in women who do not develop PE [11]. Elevated UtAPI levels in the second trimester are associated with a high risk of fetal growth restriction (FGR), PE, and/or intrauterine fetal death [12]. In the present study, a significant positive correlation was observed within the risk group (RG) between the uterine artery pulsatility index (UtAPI) values at T1 and T2. Low UtAPI MoM values at T1 remained low at T2, while elevated UtAPI MoM values at T1 were also increased at T2. The higher the UtAPI MoM value at T1, the higher it remained at T2.

Chronic hypertension and cardiovascular diseases are principal contributors to maternal and fetal/neonatal morbidity and mortality [13,14]. Studies indicate that chronic hypertension in pregnancy escalates the risk of adverse maternal outcomes [15,16]. Superimposed pre-eclampsia affects approximately 13 to 40% of pregnant individuals with chronic hypertension [17]. According to the current classification of chronic hypertension, individuals diagnosed with stage 1 hypertension during the first trimester are observed to have a two- to threefold increased risk of developing preeclampsia or any other hypertensive disorder during pregnancy, compared to those who are normotensive [18,19]. Notably, the severity of the disease tends to be less compared to those with stage 2 hypertension [20]. Poon et al. describe that the subgroup of women with pre-existing arterial hypertension before pregnancy benefits less from aspirin prophylaxis and FTS. The effect of aspirin is reduced in patients with pre-existing arterial hypertension [21].

Our results described indicate that the risk factor of pre-existing arterial hypertension significantly affects the development of uterine artery pulsatility index (UtAPI) between the two time points. Women with this risk factor exhibited a less steep decline in UtAPI MoM values and reduced responsiveness to aspirin. Consequently, women with pre-existing arterial hypertension require intensified pregnancy care.

Abnormal placental development leads to hypoperfusion, which becomes increasingly severe as pregnancy progresses. This is due to the abnormal uterine vasculature's inability to accommodate the rising blood flow demands to the fetus and placenta as gestational age increases. As pregnancy progresses, placental hypoperfusion, hypoxia, and ischemia play crucial roles in the pathogenesis of pre-eclampsia. These conditions likely stimulate the placenta to release a range of factors into the maternal bloodstream, including antiangiogenic agents like soluble fms-like tyrosine kinase-1 (sFlt-1) and endoglin [22,23]. In cases of pre-eclampsia, cytotrophoblast cells invade the decidual sections of the spiral arteries but do not extend into the myometrial segments. Consequently, the spiral arteries do not transform into the vessels typical of healthy placentation. Instead, these arteries remain constricted, leading to diminished placental blood flow and resulting in hypoxia of the trophoblast tissue. In the risk group (RG), despite aspirin prophylaxis, there were consistently higher UtAPI MoM values at both T1 and T2 compared to the control group (CG). When considering the UtAPI MoM values for both CG and RG, there was a decrease in UtAPI MoM values from T1 to T2 in both groups. Notably, the higher the UtAPI MoM value at T1, the higher it remained at T2. This relationship held true for both CG and RG. However, the RG had significantly higher UtAPI MoM values at T2 compared to the CG. The grouping of patients led to the expectation that the RG would have higher UtAPI MoM values at both T1 and T2 than the CG. These findings align with previous studies, where patients who developed pre-eclampsia had higher UtAPI values during the first and second trimesters compared to those who did not develop pre-eclampsia [21,24].

An elevated maternal BMI can be a risk factor for the development of PE [25]. The specific mechanisms by which obesity increases the risk for pre-eclampsia are not fully elucidated. However, the pathophysiological changes associated with obesity-related cardiovascular risks-such as insulin resistance and oxidative stress are also likely contributors to the elevated incidence of pre-eclampsia among obese pregnant individuals [26]. The unexpected result of a stronger decline in UtAPI MoM values from T1 to T2 within the group with elevated BMI at T1 suggests that BMI is not a definitive or informative risk factor for PE. It becomes clear that an increased BMI can be a risk factor for PE, but additional factors should be considered for PE assessment. No significant correlations were observed between uteroplacental perfusion (notching and UtAPI MoM at T1) and the HC/AC ratio at T2, nor between maternal factors such as BMI, nicotine, pre-existing arterial hypertension, and the HC/AC ratio at T2. However, a highly significant negative correlation existed between gestational weeks and the HC/AC ratio at T2 in the present study.

The abdominal circumference is one of the earliest affected parameters in impaired fetal growth. The head circumference is a stable parameter that changes minimally with growth. The HC/AC ratio reflects the discrepancy between HC and AC in cases of impaired fetal growth. As a gestational age-dependent parameter, the HC/AC ratio can be used to assess and monitor fetal growth restriction (FGR).

Comparing RG and CG, despite aspirin prophylaxis, the RG had significantly higher UtAPI MoM values in the first and second trimesters. The RG also showed an increased likelihood of presenting notching in the second trimester compared to the CG. Thus, despite aspirin prophylaxis, the RG exhibited impaired uteroplacental perfusion in the second trimester.

Minimal changes in fetal growth dynamics were observed within the RG compared to the CG. RG patients who exhibited notching at T1 had significantly smaller AC measurements below the 50th percentile at T2. In contrast, the CG showed significantly larger AC at T2 compared to the RG. Although statistical significance was not consistently observed for the associations between uteroplacental perfusion at T1 and fetal biometry at T2, the data suggests that effects and changes may become more evident during the later stages of pregnancy. The chosen time points in this study may have been too early to fully capture potential relationships between uteroplacental perfusion and fetal biometry or to assess the clinical manifestation of small-for-gestational-age (SGA) or FGR.

In the present study, the focus deliberately centered on the specific period from the first to the second trimester. We aimed to investigate whether subgroups could emerge within a positive screening collective that differs in terms of the further development of uteroplacental perfusion and can be categorized based on risk profiles. Due to the relatively large study cohort for a single-center study, we were able to statistically analyze influencing factors in detail, providing interesting and clinically relevant information for the transition from the first to the second trimester in pregnant women at risk for pre-eclampsia.

Strength and limitations

The strength of our study is the specific focus on the early development of utero-placental perfusion and its transition towards the second trimester in a two-group setting (high-risk and low-risk for PE). To our knowledge this has not been addressed in detail yet. The study has several limitations that may impact the interpretation and applicability of its findings. Firstly, the retrospective design limits the ability to control for all potential confounding variables, increasing susceptibility to selection bias compared to a prospective study design. Secondly, as a single-center study, the generalizability of the results to other populations may be limited. Differences in regional practices, patient demographics, and clinical protocols across various regions or countries could affect the applicability of the findings to other settings. Additionally, the follow-up period of the study extends only until the second trimester. This duration may be insufficient to determine whether early trends in uteroplacental perfusion accurately predict clinical outcomes at birth or in the postpartum period.

## Conclusions

This study highlights the importance of evaluating ultrasound parameters from the first to the second trimester to assess the development of uteroplacental perfusion and better estimate the risk of PE in pregnant women. The findings underscore the significant predictive value of the uterine artery pulsatility index (UtAPI) and the presence of notching in the second trimester as indicators of PE development.

The data further reveal a crucial link between pre-existing arterial hypertension and the trajectory of UtAPI values, with affected women showing a less marked reduction in these values from the first to the second trimester. This subgroup of women, especially those with persistent notching and elevated UtAPI values across both trimesters, requires closer observation throughout pregnancy due to their reduced response to aspirin prophylaxis and increased risk of adverse outcomes.

The necessity for subgroup analysis becomes evident, particularly for women with pre-existing arterial hypertension who exhibit distinct patterns in UtAPI development. Recognizing the nuanced differences within this subgroup may lead to more effective clinical management strategies, enhancing overall care and reducing potential maternal and perinatal complications The current study reinforces the value of repeated and detailed assessments throughout pregnancy, which can significantly impact clinical outcomes by enabling timely and appropriate interventions.
